# The Development of Three Long Universal Nuclear Protein-Coding Locus Markers and Their Application to Osteichthyan Phylogenetics with Nested PCR

**DOI:** 10.1371/journal.pone.0039256

**Published:** 2012-06-14

**Authors:** Xing-Xing Shen, Dan Liang, Peng Zhang

**Affiliations:** Key Laboratory of Gene Engineering of the Ministry of Education, State Key Laboratory of Biocontrol, School of Life Sciences, Sun Yat-Sen University, Guangzhou, People’s Republic of China; University of Lausanne, Switzerland

## Abstract

**Background:**

Universal nuclear protein-coding locus (NPCL) markers that are applicable across diverse taxa and show good phylogenetic discrimination have broad applications in molecular phylogenetic studies. For example, RAG1, a representative NPCL marker, has been successfully used to make phylogenetic inferences within all major osteichthyan groups. However, such markers with broad working range and high phylogenetic performance are still scarce. It is necessary to develop more universal NPCL markers comparable to RAG1 for osteichthyan phylogenetics.

**Methodology/Principal Findings:**

We developed three long universal NPCL markers (>1.6 kb each) based on single-copy nuclear genes (KIAA1239, SACS and TTN) that possess large exons and exhibit the appropriate evolutionary rates. We then compared their phylogenetic utilities with that of the reference marker RAG1 in 47 jawed vertebrate species. In comparison with RAG1, each of the three long universal markers yielded similar topologies and branch supports, all in congruence with the currently accepted osteichthyan phylogeny. To compare their phylogenetic performance visually, we also estimated the phylogenetic informativeness (PI) profile for each of the four long universal NPCL markers. The PI curves indicated that SACS performed best over the whole timescale, while RAG1, KIAA1239 and TTN exhibited similar phylogenetic performances. In addition, we compared the success of nested PCR and standard PCR when amplifying NPCL marker fragments. The amplification success rate and efficiency of the nested PCR were overwhelmingly higher than those of standard PCR.

**Conclusions/Significance:**

Our work clearly demonstrates the superiority of nested PCR over the conventional PCR in phylogenetic studies and develops three long universal NPCL markers (KIAA1239, SACS and TTN) with the nested PCR strategy. The three markers exhibit high phylogenetic utilities in osteichthyan phylogenetics and can be widely used as pilot genes for phylogenetic questions of osteichthyans at different taxonomic levels.

## Introduction

Over the past two decades, nuclear protein-coding locus (NPCL) markers have become popular tools for inferring the evolutionary history between vertebrate species at different taxonomic levels [1]–[10]. NPCL markers are based on nuclear exons, but these exons are usually short (less than 500 bp). Therefore, most NPCL markers are short and cannot provide sufficient information to resolve relationships among broadly diverged vertebrate taxa, i.e., taxa that diverged 20–420 Ma (million years ago). For example, the popular marker c-mos is often represented by very short (375 bp) fragments. In general, long markers comprise more phylogenetic signals than short ones, the resolution of resulting trees from long markers is normally higher than those from short ones. Therefore, for practical purposes, systematists are commonly willing to use some long markers (>1,000 bp) with tested good phylogenetic performance to address their questions at hand first.

RAG1 is one commonly used long NPCL marker. It takes advantage of a long (∼3 kb) and uninterrupted exon that is found across osteichthyans, has an overall evolutionary rate that is appropriate for evolutionary events from 20 to 420 Ma and furthermore, contains slightly faster- or slower-evolving regions that could resolve problems at different taxonomic levels. Due to these advantages, RAG1 has been widely used for osteichthyan phylogenetic studies, and more than 15,000 RAG1 sequence records have been deposited in the NCBI GenBank. If there are more NPCL markers like RAG1, which can be easily amplified across osteichthyans and are long enough to be phylogenetically informative at different taxonomic levels, people can use them to quickly investigate framework relationships for many taxa of interest. However, such long universal NPCL markers remain relatively limited. Therefore, it is necessary to develop more number of long universal markers suitable for osteichthyan phylogenetics. The first step toward developing these markers is to locate exons that are of the appropriate length (more than 3 kb), that are uninterrupted by introns in a diverse range of osteichthyan taxa, and that contain both fast- and slow-evolving regions.

In our previous study [10], we analyzed multiple genome alignments and developed 21 new NPCL markers for use in tetrapods. However, these markers are not long enough (normally <1,000 bp) and are difficult to be applied in bony fishes and amphibians. Therefore, we reinvestigated these 21 nuclear protein-coding genes based on a large set of genome data available from the ENSEMBL database. We found that three single-copy genes (KIAA1239, SACS and TTN) contain large exons (3.9 kb, 11.6 kb and 17.1 kb, respectively) and are fairly well conserved from ray-finned fishes to mammals. Further analyses of these exons indicated that they contain regions with variable evolutionary rates. These properties make these three nuclear genes potential candidates for long universal NPCL markers.

One of the difficulties in developing universal NPCL markers is in ensuring a high success rate of PCR amplification across divergent taxa. In general, degenerate primers are designed based on the conserved protein sequences. However, primers with high degeneracy often lack of amplification specificity, producing many non-specific amplicons or amplification failures. In contrast, primers with low degeneracy typically only work in a subset of samples due to a lack of sensitivity across diverse taxa. For example, Fong and Fujita [11] explored 75 new protein-coding genes across vertebrates and tested degenerate primers in three species, but nearly 53% of the tested fragments were not amplified successfully. Recently, nested PCR has been popular for amplifying specific sequences [1], [12]–[14]. Nested PCR is a modification of standard PCR that uses two sets of primers in two separate PCR rounds to amplify the target fragment, in which the product of the first round of PCR serves as the DNA template for the second round of PCR. The advantage of nested PCR is that it is extremely sensitive and specific when amplifying target sequences from complex genomic environments compared to standard PCR. Therefore, evaluating the technical differences between nested PCR and standard PCR will have practical implications on the application of NPCL markers and the development of universal markers.

With the advent of next generation sequencing (NGS) technologies, phylogenomic studies based on whole genome sequences or transcriptomes are becoming more and more common. Nevertheless, although the NGS-based approach is a promising way to reconstruct the tree of life, taxon sampling in such studies are normally restricted because of economic consideration and the difficulties on sample manipulations. In contrast, the conventional PCR-based method is still a more practical and cost-efficient way to generate sequences for many taxa. On the other hand, considering the bulk of worldwide museum-preserved specimens, the PCR-based method seems to be the only solution at present to analyze those samples. In this study, our goal is to increase the number of long universal NPCL markers comparable to RAG1 that can be used as "standard and pilot" markers for quick phylogenetic investigations among osteichthyans at different taxonomic levels. We investigated the phylogenetic utility of three long NPCL markers (KIAA1239, SACS and TTN) together with the reference marker RAG1 among osteichthyans. Meanwhile, we compared the ease of amplification of the three new NPCL markers in nested PCR and standard PCR. We showed that these three long NPCL markers are useful tools for phylogenetic studies of osteichthyans at broad taxonomic levels and that the nested PCR strategy is much more sensitive and specific than the conventional PCR strategy.

## Results

### General Features of the Large Exons in the Four Marker Genes

The lengths of the large exons in RAG1, KIAA1239, SACS, and TTN that were used for the development of NPCL markers are approximately 3 kb, 3.9 kb, 11.6 kb and 17.1 kb, respectively. These exons are not interrupted by introns in any of the 16 osteichthyan species observed. The conservation profile for each exon is described by the conservation diagram presented in Figure S1. Regions with a high density of long black bars indicate that the genetic divergence across the given taxa is low and that sequences are highly similar across species. For the reference gene RAG1, the conservation profile across the whole exon is not uniform; the first third of the gene (20∼1,200 bp) is fairly variable (Fig. S1). In contrast, KIAA1239, SACS and TTN are more uniform (Fig. S1), indicating that these exons are more suitable for the development of universal NPCL markers. In addition, we evaluated the overall mean distances (evolutionary rates) for each of four exon alignments in MEGA 5 [15]. The overall mean distances of these three exons are similar to each other (0.295 in KIAA1239, 0.333 in SACS, 0.327 in TTN), while RAG1 has a higher value, 0.423. However, if the fast-evolving forward third region of the RAG1 exon is removed, the overall mean distances of RAG1 decrease to 0.316, similar to the values measured for the three new genes.

### Summary of PCR Amplifications

A total of 14 primer pairs were used to amplify the four long NPCL markers: 10 were newly designed, and 4 were published in previous studies [10], [16]. The lengths of the target PCR fragments ranged from 890 to 1,210 bp. We successfully obtained a 1,488-bp fragment for RAG1, a 1,737-bp fragment for KIAA1239, a 2,211-bp fragment for SACS and a 1,698-bp fragment for TTN. Newly generated sequences were deposited in GenBank under accession numbers JN979993–JN980079, JQ929565–JQ929580 (see Table S1).

**Figure 1:**
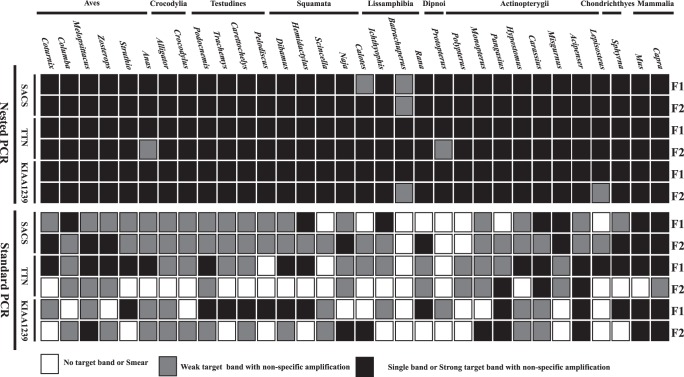
Comparison amplification efficiency between nested PCR and standard PCR for three long NPCL markers. Each long NPCL marker was amplified in two contiguous and overlapping fragments (F1 and F2). Three different color cells are used to represent the agarose gel electrophoretic images of PCR products. For complete species names, please refer to Table S1.

**Figure 2:**
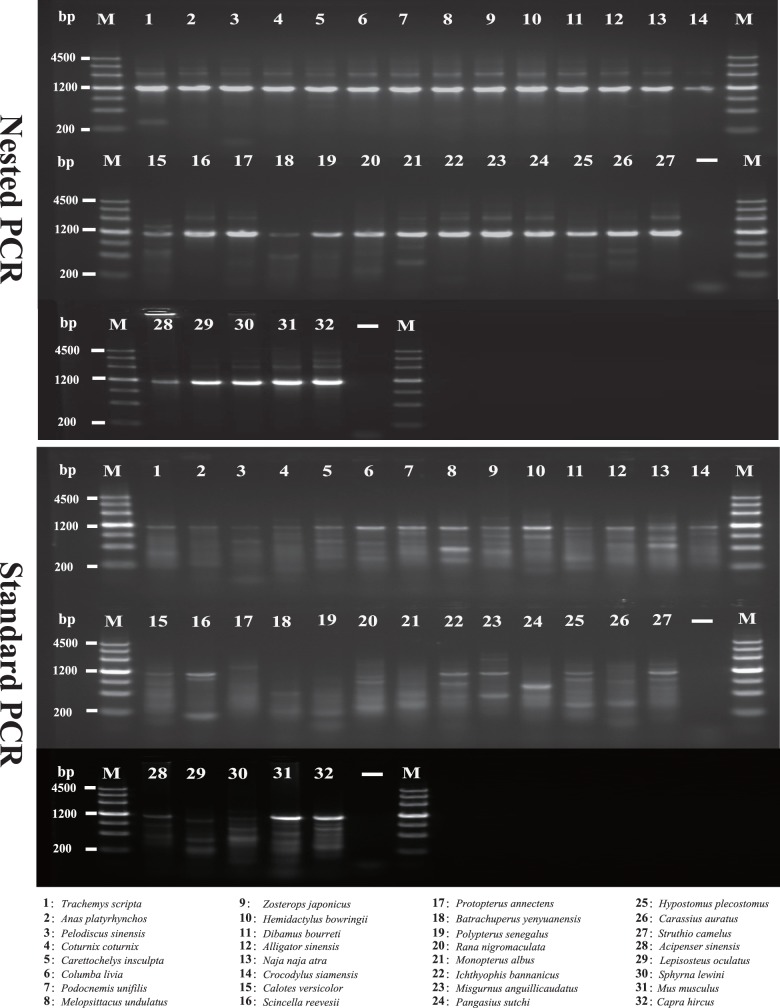
Agarose gel electrophoretic analysis of the PCR products. The first fragments of the long NPCL marker SACS (SACS-F1) were amplified in 32 taxa using nested PCR and standard PCR, respectively. The upper image shows the results of nested PCR amplifications, and the lower image shows the results of standard PCR amplifications. Lanes 1–32 show identical PCR amplifications performed in different species. “-”: negative controls, “M”: DNA ladder.

To compare the nested PCR and standard PCR strategies, we separately amplified two overlapping fragments for each of three long NPCL markers using both PCR methods. The results of these PCR experiments are summarized in Figure 1. We categorized the agarose electrophoretic images of the PCR products into three groups: no target band or smear, weak target band with non-specific amplification and strong target band with non-specific amplification. Overall, the amplification success rate and efficiency of the nested PCR were overwhelmingly higher than that of the standard PCR. The PCR success rate was 100% for the nested PCR method but only 70.3% for the standard PCR method. Furthermore, the proportion of reactions yielding strong target bands with non-specific amplification in the nested PCR was notably higher than that in the standard PCR (96.4% versus 28.6%). Finally, some species (e.g., *Batrachuperus yenyuanensis, Protopterus annectens*) are somewhat refractory to target band amplification using standard PCR, but nested PCR was able to produce strong or weak target bands for these difficult samples. The experimental differences between the two PCR methods are demonstrated visually in Figure 2, which shows an agarose gel used to separate the products of amplification of the first fragments of SACS.

### Phylogenetic Analyses

**Figure 3:**
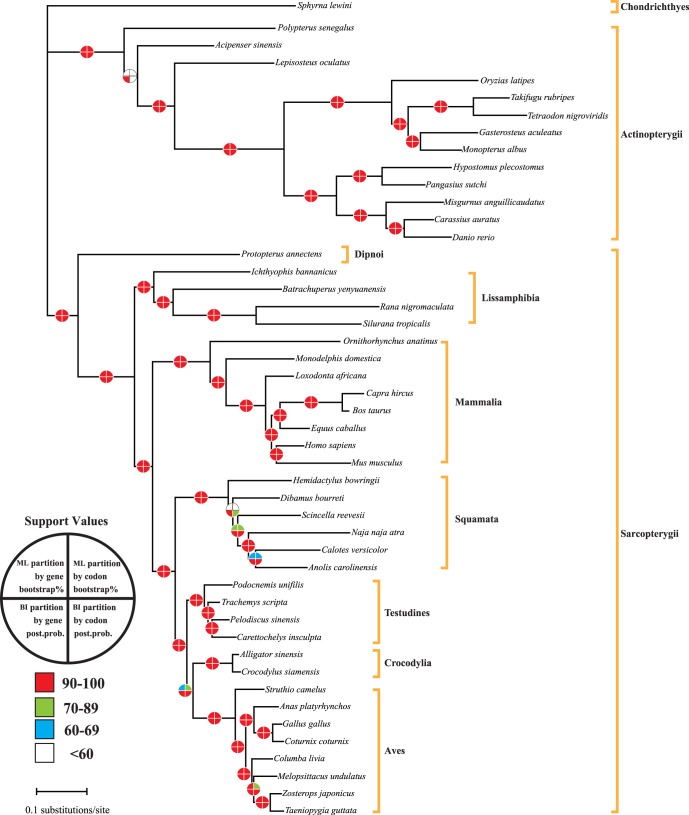
Phylogram derived from analysis of the concatenated four long NPCL markers. Phylogenetic relationships among osteichthyans were based on maximum likelihood and Bayesian inference analyses of the combined data set (7,134 bp) under 3-partition and 4-partition strategies (by codon and by gene). The two quarter circles above the branches represent the bootstrap proportions for partitioned ML analyses and the two quarter circles below branches represent the Bayesian posterior probabilities for partitioned BI analyses. Branch lengths were estimated in the 4-partition ML (by gene) analysis on a concatenated dataset (4 GTR +Γ+I models for 4 gene partitions).

The refined alignments of RAG1, KIAA1239, SACS, TTN are 1,488 nt, 1,737 nt, 2,211 nt, 1,698 nt in length, respectively. The BI and ML analyses on the concatenated dataset (7,134 nt) under the three different partitioning strategies (3-partition, 4 partition, and 12-partition) produce the same topology and similar support values. Most nodes (85%) are strongly supported with ≥95 bootstrap values (BS) and 1.0 Bayesian posterior probabilities (PP) (Fig. 3). The well-resolved tree inferred from the concatenated dataset (Fig. 3) generally agrees with the currently accepted osteichthyan phylogeny.

For each of the four NPCL markers (RAG1, KIAA1239, SACS and TTN), both partitioned BI and ML yielded almost identical trees with similar branch support values (see Figs. S2, S3, S4, S5). In all analyses, the monophyly of six animal groups (Actinopterygii, Squamata, Testudines, Aves, Crocodylia and Mammalia) are strongly supported (ML bootstrap >95% and Bayesian PP  = 1.0; Figs. S2, S3, S4, S5). The monophyly of Lissamphibia was not recovered by RAG1 (Fig. S2) but was well supported by KIAA1239 (ML bootstrap  = 95%; Fig. S3) and SACS (ML bootstrap  = 89%; Fig. S4) and weakly supported in TTN (ML bootstrap  = 50%; Fig. S5).

Phylogenetic relationships estimated from single long NPCL markers alone are generally similar to those estimated from the concatenated dataset. However, several relationships with weak support are incongruent among RAG1, KIAA1239, SACS and TTN (Figs. S2, S3, S4, S5). These conflicts are found mainly in currently uncertain relationships, such as the interrelationships within Neoaves, the early splitting Squamata, the placement of Testudines and the relationship between Acipenseriformes, Lepisosteiformes and Teleostei.

### Characteristics of the Four Long Universal NPCL Markers

**Figure 4:**
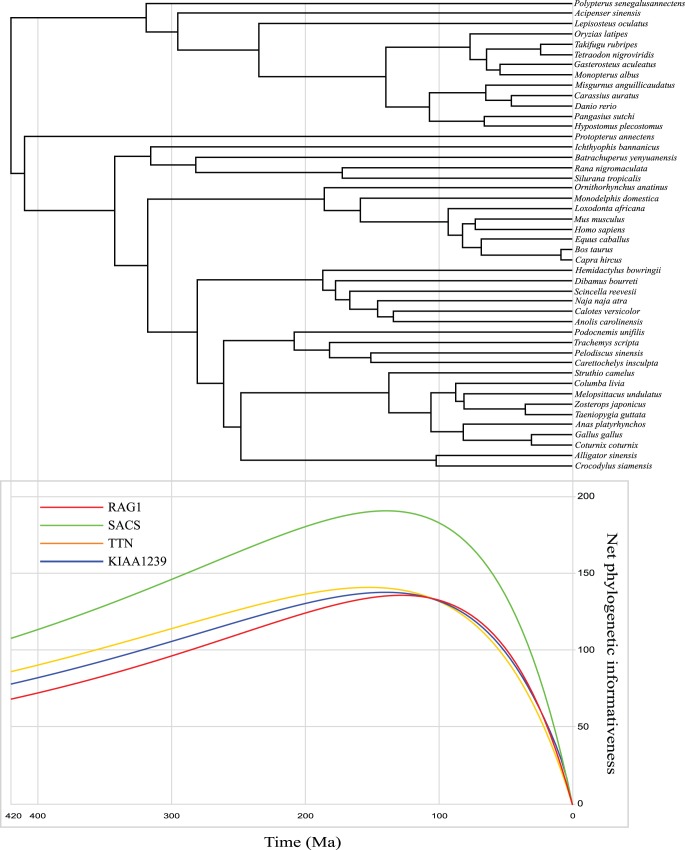
Phylogenetic informativeness (PI) profile of the reference NPCL marker RAG1 and three long NPCL markers. The timetree was newly estimated based on the concatenated dataset (7,134 bp). The PI profile was generated using the online program PhyDesign [52]. For more detail, please see the text.

Figure 4 shows the phylogenetic informativeness (PI) profile curves for the four long NPCL markers tested in this study. According to these curves, the phylogenetic performance of the three developed NPCL markers are generally comparable (or even higher; SACS) to that of RAG1. Klopfstein et al. [17] argued that estimating phylogenetic informativeness profile (PI) of the marker is needed to take cautions when including more than 4 taxa. Therefore, it is necessary to compare the information content of each marker with more indexes. We thus estimated a series of indicating parameters for each marker such as GC content, gamma shape parameter (Alpha), proportion of invariable sites (Pinvar), relative substitution rate, proportion of internal branch length (Treeness), and relative composition variability (RCV) (see Table 1). For each marker, none of the parameters have significant correlation with others. For example, KIAA1239 has the lowest alpha value, but shows high value of proportion of invariable sites. For relative substitution rate, four NPCL markers show slight variations, suggesting that they have similar evolutionary rates. Treeness is an indicator used by Phillips and Penny [18] to evaluate phylogenetic signal strength. The four NPCL markers also have similar Treeness values. In general, lower RCV value of a marker means a lower chance interfered by compositional bias. The RCV values of the three NPCL markers are lower than that of the reference marker RAG1. In brief, besides the PI profiles, all estimated characteristical parameters suggested that the three NPCL markers have potential phylogenetic performance comparable to that of RAG1.

**Table 1:** Characteristical information of the 4 NPCL markers.

Gene	Length (bp)	GC%	Alpha	Pinvar	Sub. rate	Treeness	RCV
RAG1	1,488	32.3	1.17	0.35	1.07	0.324	0.111
KIAA1239	1,737	29.6	0.94	0.30	0.95	0.359	0.096
SACS	2,211	25.9	1.02	0.29	1.06	0.320	0.092
TTN	1,698	26.4	1.01	0.25	0.91	0.356	0.070

Length, length of refined alignment; Alpha, shape parameter of the gamma distribution; Pinvar, proportion of invariable sites; Sub. Rate, relative substitution rate estimated by MrBayes; Treeness, proportion of tree distance on internal branches; RCV, relative composition variability.

## Discussion

### Three Considerations for the Development of Universal NPCL Markers

Before developing NPCL markers, researchers should first consider whether the proposed marker is a member of a gene family. When targeting genes with a large number of family members, there is a high risk of amplifying paralogous genes, which have different evolutionary histories than orthologous genes and may thus hinder correct phylogenetic inference [19]. Thus, nuclear protein-coding genes with few family members, ideally single-copy genes, are the best choices for NPCL marker candidates. In our study, the recombination-activating gene (RAG) family contains two genes (RAG1 and RAG2), but RAG1 is often recognized as a "single-copy" gene because its sequence is very different from that of RAG2. In comparison with the "single-copy" gene RAG1, none of the three nuclear genes used here have paralogs, according to a search of ENSEMBL. Therefore, these genes are suitable for the development of NPCL markers.

Recent studies [20]–[23] have suggested that gene size (e.g., alignment length) is positively correlated with the phylogenetic performance. In other words, appropriately chosen long NPCL markers are sufficient to build a reliable phylogeny [2], [6]. Moreover, if researchers develop relatively long NPCL markers, the accuracy of phylogenetic inference, particularly those based on supertree strategies will be improved. Therefore, locating large exons that are uninterrupted across diverged taxa should also be considered when developing NPCL markers.

Finally, two favorable properties (single or low-copy gene family size and large exons) alone cannot guarantee that the developed long universal NPCL markers will be useful for phylogenetic reconstruction. Researchers must also consider the phylogenetic informativeness of their markers at different taxonomic levels. Ideally, a good candidate exon for the universal NPCL marker development should contain both slowly evolving regions and fast evolving regions to provide enough information for both deep and young nodes.

### The Advantage of Nested PCR

One important criterion for NPCL markers is that they should be easily amplifiable among the taxa of interest and in other groups. The low success rate of PCR amplification, however, usually limits the range of applicability of NPCL markers. Thus, identifying a PCR strategy with high amplification efficiency is an important step toward improving the applicability of NPCL markers. Nested PCR, a modification of standard PCR, has shown to be an extremely sensitive and specific method for amplifying target sequences [1], [24]. In this study, we compared the amplification efficiency of our three long NPCL markers using nested PCR and standard PCR. Our results (Fig. 1 and Fig. 2) show that the amplification efficiency of nested PCR is apparently higher than that of standard PCR.

The nested PCR strategy has been used in previous phylogenetic studies [1], [13], [14], [24], but only for fragments that could not be amplified successfully by standard PCR. In this study, we took the nested PCR strategy as standard procedure to amplify NPCL fragments across diverse taxa from chondrichthyes to mammals. As a result, we successfully obtained all target fragments with ease, including some refractory ones in our previous study [10]. Therefore, we strongly recommend that researchers choose a nested PCR strategy rather than a standard PCR strategy when developing new phylogenetic markers or working with difficult samples.

### Implications for Osteichthyan Systematics

In general, the four independent long NPCL markers and the 4-gene concatenated datasets produced similar phylogenies for 46 tested osteichthyan species (Figs. 3, S2, S3, S4, S5). In the concatenated tree (Fig. 3), nearly all nodes are strongly supported and the interrelationships between 46 tested osteichthyans are consistent with currently accepted hypotheses. For example, our concatenated data reveals two major monophyletic clades within Teleosti: the first clade (Ostariophysi) includes members of Siluriformes and Cypriniformes, the second clade (Percomorpha) comprises members of Beloniformes, Synbranchiformes, Gasterosteiformes and Tetraodontiformes. The two major clades were also found in recent multigene studies [7], [25], [26]. Within Lissamphibia, the combined data robustly recovers a sister-group relationship between frogs and salamanders, and is in agreement with most recent studies [8], [27]–[31]. Within Mammalia, our concatenated data firmly shows that Monotremata (monotremes) is the sister group of other mammals and Proboscidea branched first within placentals. Recently, Prasad et al. [32] employed 60 megabase pairs (Mb) of genomic sequences to investigate relationships for 41 mammal species. Their results also placed Monotremata (monotremes) as sister group to other mammals and Proboscidea (Xenarthra) as basal branch of placental mammals. For the avian phylogenetic tree, recent molecular studies have consistently pointed out that Aves are divided into three major Superorders: Palaeognathae, Galloanserae and Neoaves [2], [33], [34] and Palaeognathae are basal group in avian phylogeny. Our results also confirm this relationship. Besides the interrelationships found among turtle species are also consistent with the well-resolved phylogeny of extant turtles based on analyses of single mitochondrial or nuclear gene, mitochondrial genomes and concatenated datasets [35]–[38].

The concatenated tree also have several nodes without strong support (Fig. 3) that reflect currently uncertain relationships as discussed below.

Within Neoaves, the concatenated data is unable to give a decisive relationship among Columbiformes, Psittaciformes and Passeriformes (Fig. 3). Indeed, the relationships between these avian lineages were also found to be controversial in recent nuclear and mitochondrial phylogenomic studies [34], [39], [40]. Hackett et al. [34] and Wang et al. [40] reported that Passeriformes and Psittaciformes were sister groups with respect to Columbiformes based on 19 and 30 nuclear loci, respectively. This relationship was also found in our analyses of the concatenated dataset. However, based on an analysis of 70 mitochondrial genomes to reinvestigate the interrelationships among major Neoaves, Pacheco et al. [39] suggested that Passeriformes was not a sister group of Psittaciformes. The cause of this inconsistency is not yet clear and deserves further exploration.

Within Squamata, the resulting tree indicates that Gekkota is the most basal lineage of living squamates but with only weak support (Fig. 3). However, Vidal and Hedges [5] used 9 NPCL markers to infer the relationships among the major Squamata, and they argued that Dibamidae branched first within Squamata.

The uncertain placement of the Testudines has been debated in various molecular studies [4], [10], [41]–[44]. To date, the most powerful dataset is from our previous study [10], which analyzed 23 genes (21,137 bp) and produced a robust relationship as (turtles, (birds, crocodilians)). Our concatenated analyses also recovered the same relationship but without strong bootstrapping support.

For the major actinopterygian relationships, mitogenomic data and nuclear genes produced two different relationships between Acipenseriformes, Lepisosteiformes and Teleostei. Inoue et al. [45] employed 28 mitogenomic sequences to investigate major relationships among actinopterygians, and pointed out that Acipenseriformes and Lepisosteiformes group as "ancient fishes" clade closely related to Teleostei. However, actinopterygians phylogeny based on seven nuclear genes recognized Lepisosteiformes as close relatives of Teleostei but not sister group to Acipenseriformes [24]. Our combined analyses favor the latter hypothesis, this result raises confidence in the use of the four NPCL markers among actinopterygians.

### Summaries and Recommendations

In this study, we presented three long universal NPCL markers (KIAA1239, SACS and TTN; >1,600 bp each) with comparable or better phylogenetic performance among osteichthyans to that of the widely used RAG1. In addition, we evaluated the differences between nested PCR and standard PCR when amplifying NPCL marker fragments. The amplification success rate and efficiency of the nested PCR are overwhelmingly higher than those of standard PCR. By using the nested PCR strategy, the three long NPCL universal markers can be easily amplified in osteichthyans with a success rate of over 95%. Considering their good phylogenetic performance and high usability, these markers can be widely used as pilot genes for phylogenetic questions of osteichthyans at different taxonomic levels. For example, when handling large-scale studies with many taxa, people may quickly generate data for these pilot genes with ease to identify which nodes are more difficult to resolved, thus directing further actions.

## Materials and Methods

### Taxon Sampling and DNA Preparation

The classification and source or collection locality of the 46 osteichthyan species and one chondrichthyan outgroup species used in this study are shown in Table S1. These taxa represent eight major osteichthyan lineages (Actinopterygii, Dipnoi, Lissamphibia, Squamata, Testudines, Aves, Crocodylia and Mammalia). To repress long-branch attraction (LBA) artifacts [46], we tried to include more than one species for each major lineage Among the 47 selected taxa, public genome data were available for 16 taxa, while sequences for the remaining 31 taxa needed to be generated de novo. Total genomic DNA was extracted from ethanol-preserved tissues (liver or muscle) using the standard salt extraction protocol. All extracted genomic DNA was stored at - 20°C prior to PCR amplification. This study was performed in strict accordance with the guidelines developed by the China Council on Animal Care and Use. All animal processing procedures were approved by the Institutional Animal Care and Use Committee of Sun Yat-Sen University (permit number: 2011–023).

### The Development of Three Long NPCL Markers

The nucleotide sequences of the largest exons in RAG1, KIAA1239, SACS and TTN were retrieved from Ensembl for 16 osteichthyan species with available genome data: *Danio rerio*, *Oryzias Latipes*, *Gasterosteus aculeatus*, *Tetraodon nigroviridis*, *Takifugu rubripes*, *Silurana tropicalis*, *Anolis carolinensis*, *Gallus gallus*, *Taeniopygia guttata*, *Ornithorhynchus anatinus*, *Monodelphis domestica*, *Loxodonta africana*, *Equus caballus*, *Bos taurus*, *Mus musculus* and *Homo sapiens*. Each exon was aligned based on its translated amino acid sequence, and the subsequent alignment was used for marker development. Our goal was to develop long NPCL markers of over 1,500 bp. However, the size is too large to be sequenced from both ends. Therefore, we divided a long target region into two overlapping fragments of less than 1.3 kb each to facilitate sequencing from both ends. In addition, the strategy of using two overlapping fragments to cover a long marker can also be used to check for possible cross-contamination and to ensure data quality. This design is because although the nested PCR used in this study (see discussion above) is extremely sensitive for amplifying target sequences from small amounts of samples, it may increase the risk of cross-contamination during laboratory analyses.

We manually selected a region with an appropriate evolutionary rate for each of the three NPCL genes (KIAA1239, SACS and TTN). Each selected region was divided into two overlapping fragments (less than 1.3 kb) that have two conserved blocks flanking less conserved regions. For each target fragment, we designed primers for a nested PCR strategy. The first round PCR primers were used to amplify a longer region containing the target fragment. Because the first round of PCR is only used to increase the concentration of effective DNA templates, we designed first round primers with high degeneracy to match as many amino acid sequences as much as possible, i.e., to increase primer sensitivity. In contrast, the second round of PCR is used to amplify the target fragment, and thus, we avoided designing primers in amino acid residues with high degeneracy (e.g., L, R and S) to increase primer specificity. All of the primers used in this study are listed in Table 2.

**Table 2:** PCR primers used to amplify three long NPCL markers together with the reference marker RAG1.

Gene	Fragment	Primer	Sequence (5′→3′)	Assay	Product Size (bp)≈	Reference
						
KIAA1239*		KIAA1239F1	CARCCTTGGGTNTTYCARTGYAA	1st PCR		[10]
	F1	KIAA1239R1	ACMACAAAYTGGTCRTTRTGNGT			This study
		KIAA1239NF1	GAGCCNGAYATHTTYTTYGTNAA	2nd PCR	980	This study
		KIAA1239NR1	TTCACRAANCCMCCNGAAAAYTC			[10]
						
		KIAA1239F2	GAYGARAARTACYTNGTNGT	1st PCR		This study
	F2	KIAA1239R2	TCYTCNAGRTTYTTNARRAARTT			This study
		KIAA1239NF2	TTCCAYTGCTGGTAYGARGTNAC	2nd PCR	960	This study
		KIAA1239R1	ACMACAAAYTGGTCRTTRTGNGT			This study
						
SACS*		SACSF1	AARGARATHTGGAARACNGAYAC	1st PCR		This study
	F1	SACSR1	GCYTTNGCRTCRTCNGCRTTYTG			This study
		SACSNF1	CAYCCYGAAGGAMGNGTNGCNAA	2nd PCR	1150	This study
		SACSNR1	GCWACYTCYCKNGGDATRTC			This study
						
		SACSF2	AAYATHACNAAYGCNTGYTAYAA	1st PCR		This study
	F2	SACSR2	GCRAARTGNCCRTTNACRTGRAA			This study
		SACSNF2	TGYTAYAAYGAYTGYCCNTGGAT	2nd PCR	1210	This study
		SACSNR2	CKGTGRGGYTTYTTRTARTTRTG			This study
						
TTN*		TTNF1	TATGCTGARAAYATNGCNGGNAT	1st PCR		This study
	F1	TTNR1	CCMCCRTCAAAYARNGGYTT			This study
		TTNNF1	GATGGNMGKTGGYTNAARTGYAA	2nd PCR	940	[10]
		TTNNR1	AGRTCRTANACNGGYTTYTTRTT			[10]
						
		TTNF2	TAYATYGTNGARAARCGNGARAC	1st PCR		This study
	F2	TTNR2	TCRCCWGWNACYCTRAARTARTA			This study
		TTNNF2	GGYAAYGARTAYRTHTTYAGRGT	2nd PCR	1070	This study
		TTNNR2	GCWCCWCCNTCRTTNTCNGG			This study
						
**RAG1**	F1	RAG1F1	AGCTGCAGYCARTACCAYAARATGTA	Standard PCR	980	[10], [16]
		RAG1R1	AACTCAGCTGCATTKCCAATRTCACA			[10], [16]
						
	F2	RAG1F2	ACAGGATATGATGARAAGCTTGT	Standard PCR	890	[10], [16]
		RAG1R2	TTRGAGGTGTAGAGCCARTGRTGYTT			[10]
						

R = A+G; Y = C+T; W = A+T; M = A+C; K = G+T; D = A+T+G; H = A+C+T; N = A+G+C+T.

Each long NPCL marker is amplified in two contiguous and overlapping fragments (F1 and F2). * indicates NPCL marker that is amplified using both nested PCR and standard PCR in order to compare amplification difficulties between two PCR methods. For nested PCR, 1st PCR and 2nd PCR represent two separate runs, products of 1st PCR (no dilution) are used as amplification templates in 2nd PCR; For standard PCR, primers in 2nd PCR are used alone to amplify target fragments.

### PCR Amplification, Cloning, and Sequencing

We amplified two overlapping fragments for each of three long NPCL markers using both nested PCR and standard PCR to evaluate the experimental differences between the two PCR methods. Each pair of PCR primers was tested in 25-µL reaction volumes with ExTaq DNA polymerase (Takara, Dalian). Negative controls were also included in every PCR to monitor possible cross-contamination. For the nested PCR, two separate runs (first PCR and second PCR) were conducted. The first round of PCR settings were as follows: an initial denaturation step of 4 min at 94°C; followed by 45 cycles of a 45 sec denaturation at 94°C, a 40 sec annealing at 45°C, and a 2 min elongation at 72°C; followed by a final 10 min elongation at 72°C. The second round of PCR used products of the first round PCR (without dilution) as DNA templates and the following cycling conditions: an initial denaturation step of 4 min at 94°C; 35 cycles of a 45 sec denaturation at 94°C, a 40 sec annealing at 45°C, and a 2 min elongation at 72°C; followed by a final 10 min elongation at 72°C. For the standard PCR, primers from the second round of PCR were used alone to amplify target fragment from genomic DNA, and the reaction cycling settings were the same as those used for the second round of PCR.

The target PCR bands were purified by gel extraction and subsequently cloned into a PMD19-T vector (Takara, Dalian). Recombinant clones were identified by colony PCR. The resulting PCR products (at least two) were purified with ExoSap and sequenced in both forward and reverse directions with an ABI3730 DNA sequencer. All sequences were confirmed as the correct target fragments by BLAST search against the human genome. Finally, the two overlapping fragments for each NPCL marker were assembled into a contiguous fragment. No conflicts were observed within overlapping regions, indicating that the two fragments were correctly generated from the same species in all cases.

### Phylogenetic Analyses

All four NPCL markers (RAG1, KIAA1239, SACS, and TTN) were aligned using the G-INS-i method from MAFFT [47], [48] under the default settings according to their translated amino acid sequences. Because these genes were well aligned, alignment refinements were done manually with MEGA 5 [15]. Finally, five DNA datasets (four independent alignments and one concatenated alignment) were prepared for phylogenetic analyses. The five datasets were separately analyzed with both maximum likelihood (ML) and Bayesian inference (BI) methods under partitioned strategies. For each of the four NPCL markers, we partitioned the dataset by codon (3 partitions). For the concatenated alignment (7,134 bp), we utilized three partitioning strategies. The first strategy used 3 partitions (one partition for each codon position); the second used 4 partitions (one partition for each gene); and the third used 12 partitions (codon position partitioning across four genes). The partitioned maximum likelihood analyses (-q option) were conducted using RAxML version 7.2.6 [49]. We used the GTR+Γ+I model for each partition. A search that combined 100 separate maximum likelihood searches was applied to find the optimal tree (-f d option), and branch support for each node was evaluated with 500 rapid bootstrapping replicates (-f a option) implemented in RAxML. The partitioned Bayesian inference was conducted in MrBayes 3.2 [50]. The best-fitting model for each partitioned dataset was estimated with MrModelTest2.3 [51] using the Akaike information criterion (AIC). Nearly all partitions favored the GTR+Γ+I model, except the first codon position in the TTN, which favored the GTR+Γ model. Two MCMC runs (Unlink Revmat = (all) Statefreq = (all) Shape = (all) Pinvar = (all)) were performed with one cold chain and three heated chains (temperature set to 0.2) for 3 million generations and sampled every 100 generations. The chain stationarity was visualized by plotting -lnL against the generation number using Tracer version 1.4 (http://evolve.zoo.ox.ac.uk/beast/help/Tracer), and the first 15–50% of generations were discarded. Topologies and posterior probabilities were estimated from the remaining generations. Two runs for each analysis were compared for congruence.

### Estimating the Phylogenetic Informativeness of the Four Long NPCL Markers

To compare phylogenetic performance more clearly, we generated phylogenetic informativeness (PI) profiles of the four long NPCL markers using the online program PhyDesign (http://phydesign.townsend.yale.edu/) [52]. An ultrametric tree file and an alignment were required for estimating phylogenetic informativeness. For the ultrametric tree, we estimated divergence times with MultiDivTime [53]. The ML tree from the concatenated DNA alignment was used as the reference tree. Chondrichthyes were used as the outgroup, and the Actinopterygii–Sarcopterygii split was regarded as the ingroup root. Here, we used twelve calibration nodes. One was Actinopterygii–Sarcopterygii split (416–422 Ma) [54]; others were the same as those used in our previous study [10]. We used the gene-partitioned concatenated DNA alignment as the input alignment. The site rate estimation was based on the time reversible model conducted in HyPhy [55], following the recommendations of Lopez-Giraldez and Townsend [52].

## Supporting Information

Figure S1 **Diagram of the nucleotide alignments from the largest exons in four marker genes.** The nucleotide sequences of the largest exons in RAG1, KIAA1239, SACS and TTN are retrieved from Ensembl for 16 osteichthyan species with available genome. Exon location is referenced to the human genome, and the number in parentheses indicates the length of the nucleotide alignment. Arrows represent the locations and orientations of the PCR primers used in this study. In conservation profiles, nucleotide sequences that are identical in the same column are noted by a long black bar, those that are similar in the same column are given a short black bar, and those that are totally different are given a white bar. Detailed alignments of these four sequences are available upon request.
(EPS)

Figure S2 **Phylogram derived from analysis of the reference NPCL marker RAG1.** Phylogenetic relationships among osteichthyans were inferred from codon-partitioned maximum likelihood and Bayesian inference analyses using the reference NPCL marker RAG1 (1,488 bp). The numbers closest to the nodes are ML bootstrap proportions, followed by BI posterior probabilities. Branch lengths are based on the codon-partitioned ML analysis (3 GTR +Γ+I models for codon position partitions). Hyphens indicate nodes that are not supported in the corresponding analyses. The hyphens apply to this and all subsequent tree figures.
(EPS)

Figure S3 **Phylogram derived from analysis of the long NPCL marker KIAA1239.** Phylogenetic relationships among osteichthyans were inferred from codon-partitioned maximum likelihood and Bayesian inference analyses using the long NPCL marker KIAA1239 (1,737 bp). The numbers close to the nodes are ML bootstrap proportions, followed by BI posterior probabilities. Branch lengths are based on the codon-partitioned ML analysis (3 GTR +Γ+I models for codon position partitions).
(EPS)

Figure S4 **Phylogram derived from analysis of the long NPCL marker SACS.** Phylogenetic relationships among osteichthyans were inferred from codon-partitioned maximum likelihood and Bayesian inference analyses using the long NPCL marker SACS (2,211 bp). The numbers close to the nodes are ML bootstrap proportions, followed by BI posterior probabilities. Branch lengths are based on the codon-partitioned ML analysis (3 GTR +Γ+I models for codon position partitions).
(EPS)

Figure S5 **Phylogram derived from analysis of the long NPCL marker TTN.** Phylogenetic relationships among osteichthyans were inferred from codon-partitioned maximum likelihood and Bayesian inference analyses using the long NPCL marker TTN (1,698 bp). The numbers close to the nodes are ML bootstrap proportions, followed by BI posterior probabilities. Branch lengths are based on the codon-partitioned ML analysis (3 GTR +Γ+I models for codon position partitions).
(EPS)

Table S1 List of all species used in this study, along with GenBank accession numbers.
(DOC)

## References

[1] Saint KM, Austin CC, Donnellan SC, Hutchinson MN (1998). C-*mos*, A Nuclear Marker Useful for Squamate Phylogenetic Analysis.. Mol Phylogenet Evol.

[2] Groth JC, Barrowclough GF (1999). Basal divergencies in birds and the phylogenetic utility of the nuclear RAG-1 gene.. Mol Phylogenet Evol.

[3] Murphy WJ, Eizirik E, Johnson WE, Zhang YP, Ryder OA (2001). Molecular phylogenetics and the origins of placental mammals.. Nature.

[4] Iwabe N, Hara Y, Kumazawa Y, Shibamoto K, Saito Y (2005). Sister group relationship of turtles to the bird-crocodilian clade revealed by nuclear DNA-coded proteins.. Mol Biol Evol.

[5] Vidal N, Hedges SB (2005). The phylogeny of squamate reptiles (lizards, snakes, and amphisbaenians) inferred from nine nuclear protein-coding genes.. Comptes Rendus Biologies.

[6] Hugall AF, Foster R, Lee MSY (2007). Calibration choice, rate smoothing, and the pattern of tetrapod diversification according to the long nuclear gene RAG-1.. Syst Biol.

[7] Li C, Ortí G, Zhang G, Lu G (2007). A practical approach to phylogenomics: the phylogeny of ray-finned fish (Actinopterygii) as a case study.. BMC Evol Biol.

[8] Roelants K, Gower DJ, Wilkinson M, Loader SP, Biju SD (2007). Global patterns of amphibian diversification in the history of modern amphibians.. Proc Nat Acad Sci U S A.

[9] Townsend TM, Alegre RE, Kelley ST, Wiens JJ, Reeder TW (2008). Rapid development of multiple nuclear loci for phylogenetic analysis using genomic resources: an example from squamate reptiles.. Mol Phylogenet Evol.

[10] Shen XX, Liang D, Wen JZ, Zhang P (2011). Multiple Genome Alignments Facilitate Development of NPCL Markers: A Case Study of Tetrapod Phylogeny Focusing on the Position of Turtles.. Mol Biol Evol.

[11] Fong JJ, Fujita MK (2011). Evaluating phylogenetic informativeness and data-type usage for new protein-coding genes across Vertebrata.. Mol Phylogenet Evol.

[12] Dar SA, Kuenen JG, Muyzer G (2005). Nested PCR-denaturing gradient gel electrophoresis approach to determine the diversity of sulfate-reducing bacteria in complex microbial communities.. Appl Environ Microbiol.

[13] Karanth KP, Singh L, Collura RV, Stewart CB (2008). Molecular phylogeny and biogeography of the langurs and leaf monkeys of South Asia (Primates : Colobinae).. Mol Phylogenet Evol.

[14] Oliveira C, Avelino GS, Abe KT, Mariguela TC, Benine RC (2011). Phylogenetic relationships within the speciose family Characidae (Teleostei: Ostariophysi: Characiformes) based on multilocus analysis and extensive ingroup sampling.. BMC Evol Biol.

[15] Tamura K, Peterson D, Peterson N, Stecher G, Nei M (2011). MEGA5: molecular evolutionary genetics analysis using maximum likelihood, evolutionary distance, and maximum parsimony methods.. Mol Biol Evol.

[16] San Mauro D, Gower DJ, Oommen OV, Wilkinson M, Zardoya R (2004). Phylogeny of caecilian amphibians (Gymnophiona) based on complete mitochondrial genomes and nuclear RAG1.. Mol Phylogenet Evol.

[17] Klopfstein S, Kropf C, Quicke DLJ (2010). An evaluation of phylogenetic informativeness profiles and the molecular phylogeny of Diplazontinae (Hymenoptera, Ichneumonidae).. Syst Biol.

[18] Phillips MJ, Penny D (2003). The root of the mammalian tree inferred from whole mitochondrial genomes.. Mol Phylogenet Evol.

[19] Koonin EV (2005). Orthologs, paralogs, and evolutionary genomics.. Annu Rev Genet.

[20] Rokas A, Williams BL, King N, Carroll SB (2003). Genome-scale approaches to resolving incongruence in molecular phylogenies.. Nature.

[21] Ciccarelli FD, Doerks T, von Mering C, Creevey CJ, Snel B (2006). Toward automatic reconstruction of a highly resolved tree of life.. Science.

[22] Rasmussen MD, Kellis M (2007). Accurate gene-tree reconstruction by learning gene- and species-specific substitution rates across multiple complete genomes.. Genome Res.

[23] Aguileta G, Marthey S, Chiapello H, Lebrun MH, Rodolphe F (2008). Assessing the performance of single-copy genes for recovering robust phylogenies.. Syst Biol.

[24] Kikugawa K, Katoh K, Kuraku S, Sakurai H, Ishida O (2004). Basal jawed vertebrate phylogeny inferred from multiple nuclear DNA-coded genes.. BMC Biol.

[25] Steinke D, Salzburger W, Meyer A (2006). Novel Relationships Among Ten Fish Model Species Revealed Based on a Phylogenomic Analysis Using ESTs.. Journal of Molecular Evolution.

[26] Mayden RL, Chen W-J, Bart HL, Doosey MH, Simons AM (2009). Reconstructing the phylogenetic relationships of the earth’s most diverse clade of freshwater fishes–order Cypriniformes (Actinopterygii: Ostariophysi): a case study using multiple nuclear loci and the mitochondrial genome.. Mol Phylogenet Evol.

[27] Zhang P, Zhou H, Chen YQ, Liu YF, Qu LH (2005). Mitogenomic perspectives on the origin and phylogeny of living amphibia.. Syst Biol.

[28] Frost DR, Grant T, Faivovich J, Bain RH, Haas A (2006). The amphibian Tree of Life.. Bull Am Mus Nat Hist.

[29] Zhang P, Wake DB (2009). Higher-level salamander relationships and divergence dates inferred from complete mitochondrial genomes.. Mol Phylogenet Evol.

[30] Pyron RA, Wiens JJ (2011). A large-scale phylogeny of Amphibia including over 2800 species, and a revised classification of extant frogs, salamanders, and caecilians.. Mol Phylogenet Evol.

[31] San Mauro D (2010). A multilocus timescale for the origin of extant amphibians.. Mol Phylogenet Evol.

[32] Prasad AB, Allard MW, Comparative N, Program S, Green ED (2008). Confirming the Phylogeny of Mammals by Use of Large Comparative Sequence Data Sets.. Mol Biol Evol.

[33] Sorenson MD, Oneal E, Garc J, Mindell DP, Garcia-Moreno J (2003). More taxa, more characters: the hoatzin problem is still unresolved.. Mol Biol Evol.

[34] Hackett S, Kimball R, Reddy S, Bowie R, Braun E (2008). A phylogenomic study of birds reveals their evolutionary history.. Science.

[35] Shaffer HB, Meylan P, McKnight ML (1997). Tests of turtle phylogeny: molecular, morphological, and paleontological approaches.. Syst Biol.

[36] Krenz JG, Naylor GJP, Shaffer HB, Janzen FJ (2005). Molecular phylogenetics and evolution of turtles.. Mol Phylogenet Evol.

[37] Parham JF, Feldman CR, Boore JL (2006). The complete mitochondrial genome of the enigmatic bigheaded turtle (*Platysternon*): description of unusual genomic features and the reconciliation of phylogenetic hypotheses based on mitochondrial and nuclear DNA.. BMC Evol Biol.

[38] Thomson RC, Shedlock AM, Edwards SV, Shaffer HB (2008). Developing markers for multilocus phylogenetics in non-model organisms: A test case with turtles.. Mol Phylogenet Evol.

[39] Pacheco MA, Battistuzzi FU, Lentino M, Aguilar R, Kumar S (2011). Evolution of modern birds revealed by mitogenomics: timing the radiation and origin of major orders.. Mol Biol Evol.

[40] Wang N, Braun EL, Kimball RT (2012). Testing Hypotheses about the Sister Group of the Passeriformes Using an Independent 30-Locus Data Set.. Mol Biol Evol.

[41] Hedges SB, Poling LL (1999). A molecular phylogeny of reptiles.. Science.

[42] Cao Y, Sorenson MD, Kumazawa Y, Mindell DP, Hasegawa M (2000). Phylogenetic position of turtles among amniotes: evidence from mitochondrial and nuclear genes.. Gene.

[43] Zardoya R, Meyer A (1998). Complete mitochondrial genome suggests diapsid affinities of turtles.. Proc Nat Acad Sci U S A.

[44] Zardoya R, Meyer A (2001). The evolutionary position of turtles revised.. Naturwissenschaften.

[45] Inoue JG, Miya M, Tsukamoto K, Nishida M (2003). Basal actinopterygian relationships: a mitogenomic perspective on the phylogeny of the “ancient fish”. Mol Phylogenet Evol.

[46] Bergsten J (2005). A review of long-branch attraction.. Cladistics.

[47] Katoh K, Misawa K, Kuma K, Miyata T (2002). MAFFT: a novel method for rapid multiple sequence alignment based on fast Fourier transform.. Nucleic Acids Res.

[48] Katoh K, Kuma K, Toh H, Miyata T (2005). MAFFT version 5: improvement in accuracy of multiple sequence alignment.. Nucleic Acids Res.

[49] Stamatakis A (2006). RAxML-VI-HPC: maximum likelihood-based phylogenetic analyses with thousands of taxa and mixed models.. Bioinformatics.

[50] Ronquist F, Teslenko M, Mark van der P, Ayres D, Darling A (2012). MrBayes 3.2: Efficient Bayesian phylogenetic inference and model choice across a large model space.. Syst Biol.

[51] Nylander JAA (2004). MrModeltest v2.0. Program distributed by the author. Evolutionary Biology Centre, Uppsala University.. http://www.ebc.uu.se/systzoo/staff/nylander.html/.

[52] Lopez-Giraldez F, Townsend JP (2011). PhyDesign: a webapp for profiling phylogenetic informativeness.. BMC Evol Biol.

[53] Thorne JL, Kishino H (2002). Divergence time and evolutionary rate estimation with multilocus data.. Syst Biol.

[54] Benton MJ, Donoghue PCJ, Asher RJ, Hedges SB, Kumar S (2009). Calibrating and constraining molecular clocks..

[55] Pond SL, Frost SD, Muse SV (2005). HyPhy: hypothesis testing using phylogenies.. Bioinformatics.

