# Supplementary material for: The Development of Three Long Universal Nuclear Protein-Coding Locus Markers and Their Application to Osteichthyan Phylogenetics with Nested PCR
Source: PLoS One. 2012 Jun 14;7(6):e39256. doi: 10.1371/journal.pone.0039256 (PMC3375249; doi:10.1371/journal.pone.0039256)
Supplement: Table S1 — List of all species used in this study, along with GenBank accession numbers. (DOC) [file pone.0039256.s006.doc]

| **Taxonomy** | | **Species** | **Source or collection locality** | **GenBank accession number** | | | |
| --- | --- | --- | --- | --- | --- | --- | --- |
|  | |  |  | **RAG1** | **KIAA1239** | **SACS** | **TTN** |
| Mammalia | Primates | *Homo sapiens* | Public Genome Project  Ensembl | Ensembl | Ensembl | Ensembl | Ensembl |
|  | Rodentia | *Mus musculus* | Public Genome Project | Ensembl | Ensembl | Ensembl | Ensembl |
|  | Perissodactyla | *Equus caballus* | Public Genome Project | Ensembl | Ensembl | Ensembl | Ensembl |
|  | Artiodactyla | *Bos taurus* | Public Genome Project | Ensembl | Ensembl | Ensembl | Ensembl |
|  | Artiodactyla | *Capra hircus* | Commercial food source | JQ929570 | JQ929566 | JQ929574 | JQ929578 |
|  | Proboscidea | *Loxodonta africana* | Public Genome Project | Ensembl | Ensembl | Ensembl | Ensembl |
|  | Marsupialia | *Monodelphis domestica* | Public Genome Project | Ensembl | Ensembl | Ensembl | Ensembl |
|  | Monotremata | *Ornithorhynchus anatinus* | Public Genome Project | Ensembl | Ensembl | Ensembl | Ensembl |
| Aves | Struthioniformes | *Struthio camelus* | Commercial food source | AF143727 | JN980013 | JN980040 | JN980067 |
|  | Anseriformes | *Anas platyrhynchos* | Commercial food source | HQ902532 | JN980006 | JN980033 | JN980060 |
|  | Galliformes | *Gallus gallus* | Public Genome Project | Ensembl | Ensembl | Ensembl | Ensembl |
|  |  | *Coturnix coturnix* | Commercial food source | JN979998 | JN980020 | JN980047 | JN980074 |
|  | Columbiformes | *Columba livia* | Commercial food source | AY228768 | JN980016 | JN980043 | JN980070 |
|  | Psittaciformes | *Melopsittacus undulatus* | Pet trade | DQ143354 | JN980014 | JN980041 | JN980068 |
|  | Passeriformes | *Taeniopygia guttata* | Public Genome Project | Ensembl | Ensembl | Ensembl | Ensembl |
|  |  | *Zosterops japonicus* | Pet trade | FJ358145 | JN980003 | JN980030 | JN980057 |
| Crocodylia | Alligatoridae | *Alligator sinensis* | Alligator breeding center, Xuancheng, Anhui, China | AF143724 | JN979999 | JN980026 | JN980053 |
|  | [Crocodylinae](http://www.ncbi.nlm.nih.gov/Taxonomy/Browser/wwwtax.cgi?mode=Tree&id=8494&lvl=3&lin=f&keep=1&srchmode=1&unlock) | *Crocodylus siamensis* | Commercial food source | HQ902531 | JN980002 | JN980029 | JN980056 |
| Testudines | Podocnemididae | *Podocnemis unifilis* | Private captivity | HQ902538 | JN980022 | JN980049 | JN980076 |
|  | Emydidae | *Trachemys scripta* | Commercial food source | AY687915 | JN980018 | JN980045 | JN980072 |
|  | Carettochelyidae | *Carettochelys insculpta* | Private captivity | AY687904 | JN980015 | JN980042 | JN980069 |
|  | [Trionychidae](http://www.ncbi.nlm.nih.gov/Taxonomy/Browser/wwwtax.cgi?mode=Undef&id=34907&lvl=3&lin=f&keep=1&srchmode=1&unlock) | *Pelodiscus sinensis* | Commercial food source | GU085678 | JN980025 | JN980052 | JN980079 |
| Squamata | Dibamidae | *Dibamus bourreti* | Hongkong, China | HQ902539 | JN980004 | JN980031 | JN980058 |
|  | Gekkonidae | *Hemidactylus bowringii* | Guangzhou, Guangdong, China | HQ902534 | JN980008 | JN980035 | JN980062 |
|  | [Scincidae](http://www.ncbi.nlm.nih.gov/Taxonomy/Browser/wwwtax.cgi?mode=Undef&id=66056&lvl=3&lin=f&keep=1&srchmode=1&unlock) | *Scincella reevesii* | Guangzhou, Guangdong, China | HQ902536 | JN980023 | JN980050 | JN980077 |
|  | Serpentes | *Naja naja atra* | Shaoguan, Guangdong, China | HQ902537 | JN980024 | JN980051 | JN980078 |
|  | Iguania | *Anolis carolinensis* | Public Genome Project | Ensembl | Ensembl | Ensembl | Ensembl |
|  |  | *Calotes versicolor* | Guangzhou, Guangdong, China | JN979993 | JN980001 | JN980028 | JN980055 |
| Lissamphibia | Gymnophiona | *Ichthyophis bannanicus* | Beiliu, Guangxi, China | HQ902530 | JN980000 | JN980027 | JN980054 |
|  | Caudata | *Batrachuperus yenyuanensis* | Xichang, Sichuan, China | HQ902535 | JN980021 | JN980048 | JN980075 |
|  | [Anura](http://www.ncbi.nlm.nih.gov/Taxonomy/Browser/wwwtax.cgi?mode=Undef&id=8342&lvl=3&keep=1&srchmode=1&unlock) | *Silurana tropicalis* | Public Genome Project | Ensembl | Ensembl | Ensembl | Ensembl |
|  |  | *Rana nigromaculata* | Guilin, Guangxi, China | HQ902533 | JN980017 | JN980044 | JN980071 |
| Dipnoi | Protopteridae | *Protopterus annectens* | Private captivity | AY442928 | JN980012 | JN980039 | JN980066 |
| Actinopterygii | Polypteriformes | *Polypterus senegalus* | Private captivity | JN979994 | JN980005 | JN980032 | JN980059 |
|  | Acipenseriformes | *Acipenser sinensis* | Pet trade | JQ929572 | JQ929568 | JQ929576 | JQ929580 |
|  | Lepisosteiformes | *Lepisosteus oculatus* | Pet trade | JQ929569 | JQ929565 | JQ929573 | JQ929577 |
|  | [Percomorpha](http://www.ncbi.nlm.nih.gov/Taxonomy/Browser/wwwtax.cgi?mode=Undef&id=32485&lvl=3&keep=1&srchmode=1&unlock) | *Takifugu rubripes* | Public Genome Project | Ensembl | Ensembl | Ensembl | Ensembl |
|  |  | *Tetraodon nigroviridis* | Public Genome Project | Ensembl | Ensembl | Ensembl | Ensembl |
|  |  | *Gasterosteus aculeatus* | Public Genome Project | Ensembl | Ensembl | Ensembl | Ensembl |
|  |  | *Oryzias latipes* | Public Genome Project | Ensembl | Ensembl | Ensembl | Ensembl |
|  |  | *Monopterus albus* | Commercial food source | JN979995 | JN980007 | JN980034 | JN980061 |
|  | Ostariophysi | *Danio rerio* | Public Genome Project | Ensembl | Ensembl | Ensembl | Ensembl |
|  |  | *Pangasius sutchi* | Private captivity | JN979996 | JN980010 | JN980037 | JN980064 |
|  |  | *Hypostomus plecostomus* | Private captivity | JN979997 | JN980019 | JN980046 | JN980073 |
|  |  | *Carassius auratus* | Pet trade | EF186007 | JN980009 | JN980036 | JN980063 |
|  |  | *Misgurnus anguillicaudatus* | Commercial food source | AB531306 | JN980011 | JN980038 | JN980065 |
| Chondrichthyes | Carcharhiniformes | *Sphyrna lewini* | Private captivity | JQ929571 | JQ929567 | JQ929575 | JQ929579 |

TABLE S1. List of all species used in this study, along with GenBank accession numbers.
